# Barriers and Facilitators toward HIV Testing and Health Perceptions among African-American Men Who Have Sex with Women at a South Side Chicago Community Health Center: A Pilot Study

**DOI:** 10.3389/fpubh.2016.00286

**Published:** 2017-01-03

**Authors:** Ian J. Cooke, Rohan D. Jeremiah, Nataka J. Moore, Karriem Watson, Michael A. Dixon, Gregory L. Jordan, Marcus Murray, Mary K. Keeter, Courtney M. P. Hollowell, Adam B. Murphy

**Affiliations:** ^1^Department of Urology, Northwestern University Feinberg School of Medicine, Chicago, IL, USA; ^2^School of Public Health, University of Illinois at Chicago, Chicago, IL, USA; ^3^Adler University, Chicago, IL, USA; ^4^University of Illinois at Chicago, Chicago, IL, USA; ^5^Northwestern University Feinberg School of Medicine, Chicago, IL, USA; ^6^Loyola University Stritch School of Medicine, Chicago, IL, USA; ^7^Project Brotherhood Inc., Chicago, IL, USA; ^8^Division of Urology, Cook County Health and Hospitals System, Chicago, IL, USA

**Keywords:** HIV, testing, African-American, MSW, perceptions, behaviors

## Abstract

In the United States, African-Americans’ (AAs) HIV infection rates are higher than any other racial group, and AA men who have sex with women (MSW) are a significant proportion of new cases. There is little research into AA MSW HIV/AIDS knowledge, barriers, and facilitators of HIV testing in Chicago. We enrolled a convenience sample of AA MSW from a community health clinic who completed self-administered surveys assessing HIV knowledge and testing-related barriers and facilitators. The survey was a combination of questions from several validated instruments, and additional questions were written based on key informant interviews with social scientists to tailor the questionnaire for AA men living on the South Side of Chicago. We recruited 20 AA MSW (mean age 47.4 years). Sixty-five percent had incomes <$10,000/year, 30% were insured, and 50% had post-secondary education. Despite low socioeconomic status, their HIV literacy was relatively high. The identified major barriers to testing were low perceived HIV risk, concerns over privacy, and external stigma at testing sites. Future efforts should focus on educating AA MSW on actual risk for HIV and address issues of privacy and stigma at testing sites.

## Introduction

In the United States, African-Americans (AAs) have rates of HIV infection that are higher than any other racial group. AA men are infected 3.6 times more often than the general male population, with 15,847 new diagnoses in 2013 (1). Although AA men who have sex with men (MSM) comprise 78% of new HIV diagnoses among AA men (2), AA men who have sex with women (MSW) represent nearly 25% of new HIV cases in the United States (3). Furthermore, heterosexual individuals with an annual income at or below the national poverty level are 2.2 times more likely to have HIV than individuals above the poverty line (4). In Chicago, 53% of new diagnoses were among AAs, and 15.5% were due to heterosexual contact in 2014 (5). It is crucial to understand Black male attitudes toward HIV testing in Chicago, a city that diagnoses HIV with an annual incidence of 23.3/100,000 (twice the national rate) and a prevalence that is three times the national average (1, 6).

HIV testing remains a key intervention for preventing the spread of HIV, and previous studies have identified decreases in unprotected sex among individuals who receive a positive test result (7). Although AAs are tested at increased frequency compared with other racial groups, previous studies have found that 78.7% of AAs had not been tested in the past 12 months (8). Other studies examining barriers to HIV testing have identified low perceived risk as a major deterrent to testing (9–12). Fear of testing positive has also been cited (10), and a Kaiser Family Foundation report found that 7% of AAs felt that their peers would “think less of them” if they got tested (12). Among AA MSW from New York City (NYC), fear, low perception of risk, and stigma were found to be significant barriers to testing (13). AA MSW in Chicago may have unique barriers to HIV testing, and it is known that factors such as social support and attitudes regarding sex predict different HIV testing behaviors based on geographic location for AA men (14).

Among at-risk AA men in Chicago, community health programs are important sites for the consumption of health-care services and the dissemination of HIV prevention information. Project Brotherhood is a community outreach and prevention program serving AA men in the Woodlawn neighborhood on the South Side of Chicago. Its mission is “to increase the health awareness of Black men by providing health education with a cultural and gender-specific perspective” (15). Organizations like Project Brotherhood have been implemented and studied in other locations and have been successful in improving HIV testing rates in rural community health clinics (16), but there are no clear data on the role of small community centers in an urban setting (17). Project Brotherhood is unique in that it has always served and been under the leadership of AA men that anchored within its service communities. Project Brotherhood was selected for this study because of its specific focus on AA men and its goal to address minority men’s health issues. Quantifying the impact of Project Brotherhood and its commitment to the South Side of Chicago reinforces the understanding of its culturally targeted programs for AAs.

Characterization of baseline health literacy as well as barriers and facilitators to HIV testing in this group will allow for targeted interventions to promote HIV testing in high-risk AA MSW in Chicago and in similar community health centers in other urban cities. Thus, the aim of this study was to identify specific attitudes, barriers, and facilitators toward HIV testing and health perceptions among low-income AA MSW seeking care at a community clinic in Chicago.

## Materials and Methods

### Study Sample

The study sample consisted of 24 AA men over age 18 who received medical or social services at Project Brotherhood, a clinic housed within Woodlawn Health Center. We approached men in the waiting rooms of the clinic between the hours of 1:00 p.m. and 4:00 p.m. on Thursday afternoons for 10 of the 18 weeks between December 2012 and April 2013. Over this period of time, there were over 300 AA men who were seen in the clinic. We approached 42 AA men, 22 who identified as MSW and 20 who identified as MSM. In total, 20 heterosexual men and 4 non-heterosexual men enrolled, giving us a participation rate of 57%. This study focuses on MSW, therefore, four men were excluded from data analysis because they self-identified as MSM. In the Woodlawn community area of Chicago, 87.8% of residents are AAs. Twenty seven percent of the population is low income (100–199% of Federal Poverty Line), and 32.3% of the population have incomes below the Federal Poverty Line (18).

### Procedures

Eligible men were enrolled as a convenience sample based on their attendance at Project Brotherhood or the affiliated Woodlawn Health Center. Participants completed a three page self-administered survey to assess demographic characteristics, HIV-related health literacy and perceptions, barriers and facilitators to HIV testing, condom use and condom-related behaviors, trusted sources of HIV and health information, and preferred testing sites. Survey items were developed based on questions from the HIV Knowledge Questionnaire, a validated research tool for testing HIV knowledge (19). Other survey items were culled from other studies (14, 20–22) cited in HIV testing literature. These items were then adapted for the Project Brotherhood population by a social scientist with expertise in survey methods and creation of culturally tailored surveys for AAs. A portion of questions in the survey were presented as binary yes/no, while others were presented on a 5-point Likert scale (i.e., strongly disagree, disagree, neutral, agree, or strongly agree).

#### Demographic and Descriptive Information

Percentages were used to describe categorical data, and age was described by mean and standard deviation (SD) for demographic and descriptive information. Self-rated health was scored on a 5-point Likert scale as “very poor,” “poor,” “fair,” “good,” or “very good.” Ratings of perceived health status are presented as percentages for men who rated their health as either “good” or “very good.”

#### HIV-Related Health Literacy and Perceptions

The survey item “Do you know someone with HIV?” was a binary (1 = yes, 0 = no) question. The item “Are there any foods, medications, or surgeries that can lower my risk of HIV?” was also a binary response. A multiple-choice format regarding initiating of screening was used for the item “HIV screening should begin when men become sexually active.” Options for the recommended initiation of screening included “men should be screened when they become sexually active,” “18–24,” “35–29,” “40–49,” “50–59,” and “60+.” We report the number of men that selected “when they become sexually active.” The item “How often should men get checked for HIV?” was a multiple-choice response, and options were listed as “every 6 months,” “every year,” “every 5 years,” and “only after unprotected sex outside of monogamous relationship.” Other items were scored on a 5-point Likert scale, and for those items we report percentages for men who responded with either “agree” or “strongly agree” to the listed statement.

#### Barriers and Facilitators to Testing

All items in this section were scored on a 5-point Likert scale. We report percentages for men who agreed with the listed statement and responded with either “agree” or “strongly agree.”

Questions were derived from a variety of sources from the literature; fundamental examples are detailed below:

• “HIV and AIDS are the same thing” (19)
• “A person with HIV can look and feel healthy” (19)
• “How often should people get an HIV test” (22)
• “Most people with AIDS will die from it” (19)
• “Eating healthy foods can keep a person from getting HIV” (19)
• “How likely do you think you are to get HIV in your lifetime?” (23)
• “HIV infects mainly homosexuals” (21)
• “If HIV is treated properly, an average HIV-infected person can live more than 20 years” (21)
• “People with HIV/AIDS appear to be sexually promiscuous people” (21).

The Institutional Review Board of Northwestern University and Cook County Health and Hospitals System approved the protocol. Informed consent was obtained from each participant. Compensation was provided ($15) for participants’ time. Surveys were self-administered and distributed by a research coordinator.

### Analysis

Survey data were entered into an Excel database, and demographic information and questionnaire variables were analyzed using descriptive statistics and SPSS 21.

## Results

### Demographic and Descriptive Information

The study sample was a middle age, fairly educated, yet impoverished group of men. Mean age was 47.4 years, SD 9.92, with a range of 29–66 (see Table 1). All men graduated from high school, while 50% of the participants had more than a high school education. Sixty-five percent of the respondents reported a yearly income of $10,000 or less, and 30% had no income. Additionally, 25% were married, 30% had health insurance, and 50% had a primary care physician.

**Table 1:** **Demographic and descriptive information**.

Characteristics (*n* = 20)	Mean, SD
Mean age (years, SD)	47.4 (9.92)
Characteristics (*n* = 20)	%
African-American/Black	100
Self-identified as a MSW^a^	100
High school completion	100
Has had post-secondary education^b^	50
Married	25
Self-rated health as “good” or “very good”	70
Has health insurance	30
Has a primary care physician	50
Income <$10,000 per year	65
Reported no yearly income	30

*^a^Four men were excluded as they reported having sex with men*.

*^b^Post-secondary education was defined as completing an associate’s degree or greater*.

### HIV-Related Health Literacy and Perceptions

Every man surveyed endorsed that getting tested for HIV is necessary (see Table 2). Ninety-four percent of men considered there to be a difference between HIV and AIDS, and 88% felt it was possible to live a long life with HIV. Seventy-eight percent of men supported frequent testing (at least every 6–12 months). The majority (65%) of men felt that Blacks were the racial group with the highest risk for HIV, and 89% felt that testing for HIV should begin as soon as men become sexually active. Additionally, 89% of participants noted that HIV is not a homosexual disease. Overall, the sample was modestly literate in terms of HIV-related health information.

**Table 2:** **HIV-related health literacy and perceptions**.

Knows someone who was screened for or diagnosed with HIV	33%
There are foods, medications, and surgeries that can lower my risk of HIV^a^	6%
HIV screening should begin when men become sexually active^b^	89%
Men should get checked for HIV at least every 6–12 months^b^	78%
I think it is necessary to get a HIV test^c^	100%
There is a difference between HIV and AIDS^c^	94%
A man can live a long life with HIV^c^	88%
HIV is a death sentence^c^	11%
HIV is a homosexual disease^c^	11%
Blacks are the highest risk racial group for HIV^c^	65%

*^a^Survey items written as “yes/no” questions*.

*^b^Survey items written as multiple-choice questions*.

^c^Survey items written as “strongly disagree/disagree/neutral/agree/strongly agree.” Positive responses defined as “agree” or “strongly agree.”

### Barriers and Facilitators to HIV Testing

Several barriers and facilitators to HIV testing were identified (see Table 3). Only 12% of respondents were concerned about the risks associated with HIV treatment, and only 21% were concerned about being judged as promiscuous due to HIV testing. An additional barrier included concern regarding privacy of health information among 47% of respondents. No participant felt fearful of knowing his HIV status, and only 8% felt that they were likely to get HIV.

**Table 3:** **Barriers and facilitators to HIV testing**.

There are risks with HIV treatment that I am worried about^a^	12%
I have talked about HIV and condom use with peers^a^	79%
I am fearful of knowing my HIV status^b^	0%
I am likely to get HIV^b^	8%
I am afraid of my privacy/anonymity when I go to the clinic^b^	47%
If I get tested for HIV, this means I am sexually promiscuous^b^	21%
I feel open to talking about health with family or friends^b^	71%
I feel open to talking about HIV with family or friends^b^	64%

*^a^Survey items written as “yes/no” questions*.

^b^Survey items written as “strongly disagree/disagree/neutral/agree/strongly agree.” Positive responses defined as “agree” or “strongly agree.”

In terms of facilitators, participants were fairly open to discussing health (71%), HIV (64%), and condom use (79%) with family, friends, or peers.

## Discussion

Despite significantly below average socioeconomic status as measured by income and insurance status, participants demonstrated a high level of insight into HIV screening recommendations and stated that HIV testing was necessary. This likely is due to the recruitment of men within a health and social services organization. The degree to which health literacy influences HIV testing behavior is currently unclear, but other data have shown that higher health literacy is associated with better HIV-related health behaviors and outcomes. For instance, one study found that educational attainment of high school or less is associated with decreased testing frequency (24), and among people who already have a diagnosis of HIV, there is some evidence of increased medication adherence and improved outcomes among people with higher levels of health literacy (25, 26). However, additional data suggest that low health literacy may facilitate testing. Among individuals not diagnosed with HIV, poorer health literacy has been shown to be associated with HIV test acceptance (27). Despite the overall good health literacy, 92% perceived they were at low risk for HIV even though 75% of the men were unmarried.

Even with the relatively high levels of HIV-related health literacy in our study, we identified several dominant barriers to testing including low perceived HIV risk, privacy concerns (47%), and external stigma (21%) associated with testing. Our findings are similar to those of a recent study from Bond and colleagues, which reported low perception of risk and stigma as the dominant barriers to HIV testing in Black MSW in NYC (13). In NYC, the Black MSW sample cited fear of their HIV status as a major testing barrier; however, none of the men in our sample identified fear of knowing their HIV status as a deterrent to testing (28). Furthermore, in our sample, only one respondent reported feeling they were “likely” to become infected with HIV. Low perceived risk has repeatedly been cited as a barrier to testing among high-risk groups, which was consistent with the findings from the AA MSW in our sample (9–11). In fact, a report from the Kaiser Family Foundation showed that a lack of perceived risk was the most frequently cited reason for not getting tested among AAs (12). Of note, the lack of fear of HIV status among the men in our sample may have been related to their low perceived risk. Previous studies have suggested that the presence of fear may be a deterrent to testing, but our study suggests that the absence of fear may also be a barrier.

The lack of fear of knowing one’s HIV status and low perceived risk among MSW may also reflect a lack of perceived danger in acquiring HIV from a woman. Much of the public health messaging to men regarding HIV prevention has focused on the protection of women from men who are more likely to carry and transmit HIV (29). However, this message fails to consider that men are also at risk as a result of transmission from high-risk AA women, and in some settings, women are almost as likely as men to introduce HIV into a sexual partnership (29). AA MSW may not be considering their need to protect themselves from their female partner due to a lack of concern for contracting HIV from a woman.

Uncertainty regarding privacy has previously been cited as a concern associated with testing (28). In our sample, 47% of men reported privacy concerns. This was significantly more than contrasting data showing only 9% of AAs not being tested due to worries about confidentiality (12). Other than low perceptions of risk, privacy concerns were the second most cited barrier to testing among AA MSW in our study.

Other studies have identified stigma as a major barrier to HIV testing among AAs and AA MSW (13, 28). In our study, stigma was assessed by quantifying participants’ opinions regarding HIV as a “homosexual disease” and whether HIV testing suggested sexual promiscuity. Only 11% of men thought that HIV was a “homosexual disease,” consistent with the changing epidemiology of the disease. Of these measures of stigma, the top concern associated with testing was concern about being viewed as promiscuous, which was held by 21% of respondents. Community attitudes and stigmatization of HIV testing play a substantial and opposing role in influencing an individual’s decision to test. In one study from the Kaiser Foundation, 7% of AA participants felt that peers would “think less of you” if they were to be tested for HIV, while 22% of AA participants believed that peers would “think more of you” if they decided to test. These data indicate that there may be both positive and negative social pressure for testing among AAs (12). In our sample, the high percentage of men open to discussing HIV status (64%) with friends and family is indicative that there may be decreasing stigmatization of HIV in AA men served in community programs.

### Implications for Interventions

Community sites similar to Project Brotherhood have been shown to improve health literacy among AA men with low socioeconomic status, and other interventions targeting sexual health risk behaviors among AA MSW have been shown to be effective when based in community sites such as barbershops (30). In terms of implications for future interventions, our study suggests the need for continued focus on educating AA MSW about their increased risk for HIV. In addition, future research should focus on feasibility of implementing evidence-based HIV-training programs into community-based clinics that employ a culturally tailored focus to health education and prevention. Project Brotherhood has been effective because of its understanding and ability to deliver culturally appropriate programs to AA men. The featured model of this study has shown its effectiveness in establishing a critical conversation with at-risk populations such as AAs. Furthermore, our study suggests that community centers and testing sites should make privacy a priority and develop methods that promote anonymous testing.

### Limitations

Our study’s primary limitation was its small sample size. In addition, it is based on a specific group of AA MSW receiving health-care services at Woodlawn Health Center and may not be generalizable to all settings. The mean age of our sample was 47.4 and thus does not adequately represent adolescent AA men, a population with unique risk factors for HIV acquisition requiring tailored interventions. We did not evaluate some specific HIV-related behaviors such as the date of last HIV test. Finally, our results may be biased toward men who already seek social services at a men’s health clinic.

## Conclusion

Understanding the HIV-related health characteristics of specific populations is important for the development of targeted interventions due to known geographic differences in HIV testing attitudes and behaviors (14). Our study is the first to identify baseline HIV-related health literacy and barriers to testing among low-income AA MSW in Chicago.

Despite having lower socioeconomic status and incomes as compared to Black men in the rest of the country, the men in our cohort had relatively high rates of HIV-related health literacy. Although income has long been posited to be a major factor in models describing factors related to health literacy (31), our study suggests other factors may play a role in the acquisition of HIV-related health literacy among AA MSW.

We also explored barriers to HIV testing among AA MSW. The major barriers to testing in our cohort were low perceived risk and concerns over privacy, as well as external stigma due to testing. These concerns have been consistently cited in other studies as major barriers for HIV testing among AA men in settings outside of Chicago. Future research should continue to explore the role of stigma versus positive community pressure to get tested among AAs, as well as preferential testing locales, HIV perceptions, and barriers to testing in different settings.

## Ethics Statement

This study was carried out in accordance with the recommendations of The Cook County Hospital Institutional Review Board Ethics Committee with written informed consent from all subjects. All the subjects gave written informed consent in accordance with the Declaration of Helsinki.

## Author Contributions

IC—first author, data analysis, interpretation, drafting, revision, and corresponding author; RJ—data analysis, interpretation, drafting, and revision; NM—patient recruitment, conception, study design, data analysis, drafting, and revision; KW—drafting and revision; MD and GJ—data collection and analysis; MM—design, conception and recruitment, drafting, and revision; MK—data analysis, drafting, and revision; CH—design, drafting, and revision; and AM—patient recruitment, conception, study design, data analysis, drafting, revision, and corresponding author. All the authors reviewed and edited the final draft of the manuscript before submission.

## Conflict of Interest Statement

The authors declare that the research was conducted in the absence of any commercial or financial relationships that could be construed as a potential conflict of interest.
